# The Effects of Feedback on Memory Strategies of Younger and Older Adults

**DOI:** 10.1371/journal.pone.0168896

**Published:** 2016-12-29

**Authors:** Fan Zhang, Xin Zhang, Meng Luo, Haiyan Geng

**Affiliations:** 1 School of Psychological and Cognitive Sciences, Beijing Key Laboratory of Behavior and Mental Health, Peking University, Beijing, P. R. China; 2 Department of Psychology, the Chinese University of Hong Kong, Hong Kong; 3 Department of Medicine & Therapeutics, the Chinese University of Hong Kong, Hong Kong; Center for BrainHealth, University of Texas at Dallas, UNITED STATES

## Abstract

Existing literature suggests that feedback could effectively reduce false memories in younger adults. However, it is unclear whether memory performance in older adults also might be affected by feedback. The current study tested the hypothesis that older adults can use immediate feedback to adjust their memory strategy, similar to younger adults, but after feedback is removed, older adults may not be able to maintain using the memory strategy. Older adults will display more false memories than younger adults due to a reduction in attentional resources. In Study 1, both younger and older adults adjusted gist processing and item-specific processing biases based on the feedback given (i.e., biased and objective feedback). In Study 2 after the feedback was removed, only younger adults with full attention were able to maintain the feedback-shaped memory strategy; whereas, both younger adults with divided attention and older adults had increased false memories after feedback was removed. The findings suggest that environmental support helps older adults as well as younger adults to adopt a memory strategy that demands high attentional resources, but when the support is removed, older adults can no longer maintain such a strategy.

## Introduction

Recent studies on false memory have shown that younger adults are able to transfer memory strategies shaped by previous feedback to later sessions and enhance memory accuracy [1, 2]. Specifically, in accordance with feedback emphasizing different types of processing, younger adults are able to readily switch from gist-based to verbatim-based processing or *vice versa*. However, it is still unclear whether feedback can foster older adults’ use of strategies to improve memory performance.

While aging is associated with memory declines in daily life (e.g., increased false memories), older adults are able to improve memory accuracy through environmental supports (e.g., warnings about the associative nature of testing material before study, picture materials instead of text materials, or repeated study [3, 4]). Yet, whether older adults are able to maintain memory strategies when environmental supports are removed remains unknown.

This study investigated two issues: 1) the effect of feedback on memory strategy used by older adults and 2) the maintenance of memory strategies among older adults. We conducted two experiments with the Deese—Roediger—McDermott paradigm [5] in younger and older adults.

### False Memory and Aging

It is well-documented that older adults usually exhibit more false memories than younger adults. For example, during recall, older adults are more likely to confuse related events that did not occur with those that actually did occur, which is known as “associative memory illusions” [6–8]. According to Fuzzy-Trace Theory, this type of false memory is driven by two different types of memory processing [9]. Specifically, gist processing extracts essential information of one’s experience or studied materials, and in contrast, verbatim or item-specific processing preserves the exact content and focuses on the perceptual details as well as particular features of experienced items. Item-specific processing enables better distinguishing of facts from similar or related scenarios, but places higher demand on attentional resources [10, 11]. Gist processing, on the other hand, allows for the storage of the essentials as compact event records. However, focusing on semantic association and similarities between new and experienced stimuli can lead to overlooking specific details, thus, resulting in increased false memories.

In the case of older adults, age-related functional decline of the frontal and temporal lobes results in reduced cognitive resources [12]. Therefore, older adults tend to rely on memory strategies that demand fewer attentional resources, “e.g., gist-based processing”. This leads to not only more semantic activation during encoding but also less item-specific information processing and subsequently less available for monitoring during retrieval [13, 14]. Monitoring refers to the use of retrieved information to check the source of activation when making memory decisions [15]. When item-specific processing is hindered, less distinctive information will be retrieved and used for monitoring [16, 17]. Therefore, it is difficult for individuals to screen and correctly reject the related but unstudied items from studied ones, which contributes to increases in susceptibility to associative memory illusions. However, age-related increase in associative memory illusion does not only suggest increases in memory failures but also suggest an adaptive change in processing strategy. Compared with verbatim/item-specific information, gist information is more fundamental for tasks such as comprehension, categorization, and generalization across different situations in daily life [18]. According to the Selective, Optimization and Compensation model (SOC [19, 20]), from middle to older age, primary memory goals shift from striving for gains to maintaining or preventing loss. When it comes to memory, the inevitable increase of associative memory distortions reflects the fact that preserving the essentials of the past outweighs maximizing the storage of item-specific information [21–23]. Interestingly, higher levels of associative false recognitions predict better problem solving and performance in the Remote Associates Task [24, 25]. This is probably because gist processing represents information storage and underlying processes shared by both remembering the past and imagining the future (i.e., the constructive episodic stimulation hypothesis [26, 27]). As a result, when attentional resources become limited, as in the case of natural aging [28], leaning towards a gist-based memory strategy to remember the essential information can be beneficial for older adults in trying to distinguish studied items from new ones [22].

Older adults have been found to initially prioritize gist-based processing, with environmental support such as repeated studying showing high false-recognition rates of new, related items in initial trials [29]. False recognition rates then decrease in later trials, suggesting a switch to item-specific processing. Without sufficient cognitive resources, older people have shown limited ability to initiate item-specific processing [30, 31], and when engaged in divided attention conditions, younger adults also have shown compromised item-specific processing compared to gist processing [32].

Meanwhile, sufficient environmental support has been shown to help people to process distinctiveness and reduce false recognitions. For example, using picture aids or repetitively studying materials could compensate for cognitive loss among older adults [3, 4]. This may be because environmental supports remedy the declining item-specific processing caused by limited attentional resources, even though this may not enable older adults to inhibit false memories as effectively as younger adults [33, 34]. Comparing the effects of repeated study among younger adults, older adults, and patients with probable Alzheimer’s disease (AD) showed that repeated study increased true memory in all three groups, but the decrease in false memories depended on the level of cognitive ability. While younger adults had fewer false memories with study from the beginning, older adults required more studying to accumulate item-specific information and reduce the false memories, and mild AD patients were not able to inhibit false memories across five trials [29]. Therefore, it is possible that the effect of environmental support might also vary with the availability of cognitive resource, yet few studies have addressed this issue.

### Feedback and False Memory

Providing feedback to participants on memory research is one type of environmental support. Examples of common feedback include informing participants how they did in a specific trial immediately (e.g., “correct/wrong”) or over sets of trials (e.g., “you answered 70% of the trials correctly”).

Previous research has found that immediate feedback on response accuracy significantly reduces participants’ false memories [2, 35]. For instance, Jou and Foreman compared the effects of warnings, “right/wrong” feedback, and “right/wrong” feedback with small prizes across multiple study-test trials on participants’ memory performance. The results showed that false recognition rates were lower in the two conditions with feedback, suggesting that providing feedback could be more effective than some other types of environmental support in inhibiting false memories.

Feedback can shape the recognition memory decision criteria [36, 37]. In a study by Han and Dobbins [38], approximately 70% of participants’ false alarms received false feedback of “CORRECT” in the lax condition, while 70% of misses received “CORRECT” in the strict condition. The control condition included no feedback. The results revealed that people had more false alarms (i.e., a more liberal memory decision criterion) in the lax condition and more misses (i.e., a more conservative criterion) in the strict condition. These results suggested that individuals adjusted their recognition memory decision criteria to maximize the possibility of receiving positive feedback and minimize that of negative feedback. In addition to shaping decision criteria, feedback can influence encoding strategies and help to establish specific memory goals by shaping participants’ motivation and awareness of memory goals. For example, positive feedback confirming that the participants were moving towards their goals could be especially beneficial for older adults, by increasing their motivation and goal commitment for memory [39].

It is also possible that the specific diagnostic information offered by immediate feedback could influence participants’ subsequent information encoding and retrieval [1]. However, few studies have directly tested this effect of feedback. One previous study [34] used instructions with distinctive information (e.g., *subway*, asking participants “does it travel underground?”) or semantic association (e.g., subway, “is it a means of transport?”) to manipulate encoding processing, and found that younger and older adults benefitted from both types of instructions equally well. The current study tested how feedback affects memory processing and decision criteria when feedback is used as a retrieval manipulation rather than as an instruction.

### The Present Study

As reviewed above, existing literature shows environmental support can help older adults compensate for decreased item-specific processing and reduce associative memory illusions. Few studies have investigated age-related differences in adjusting or maintaining memory processing strategies under feedback [2, 35]. In particular, we were interested in exploring how feedback may differentially influence gist and item-specific processing strategies among different age groups. By modifying the paradigm of Han and Dobbins [38], we compared the effects of biased and objective feedback on subsequent memory performance. In the objective-feedback condition (OFB), accurate feedback, “i.e., ‘correct’ / ‘incorrect’”, was provided after participants’ response in each trial; whereas, in the biased-feedback condition (BFB), “correct” was provided both when participants correctly recognized learned items (i.e., hits) and when participants incorrectly judged critical lures as learned items (i.e., false alarms). Biased feedback encourages participants to recognize both studied items and critical lures as “studied”; therefore, we expected that biased feedback would reinforce responses based on gist processing. In contrast, objective feedback may remind participants of the differences between the studied items and the critical lures and therefore will facilitate item-specific processing. By presenting a series of study-test trials with biased or objective feedback, the current study examined dynamic changes in memory processing among younger and older adults.

We conducted two experiments using the Deese—Roediger—McDermott paradigm (DRM, [5]) to address our research questions. In the DRM paradigm, several lists of words (also referred to as “associates”) are presented, and the words in each list are semantically related to a thematic word (referred to as a critical lure) that is not presented. Later in the test, participants frequently recall or recognize the critical lures as “studied” (e.g., an 80% false recognition rate [40]). Given that relying on gist processing would increase the false recognitions of critical lures, while using item-specific processing would reduce this rate [8], the DRM paradigm is well suited to examining the two types of processing.

Previous literature directly used the false recognition rate of critical lures to measure gist processing [e.g., 4], which might actually include a guessing component. We adopted the formula developed by Schacter, Israel, and Racine (1999) to measure gist processing and item-specific processing [41]. We also used two indices from signal detection theory (SDT, [42, 43]) to analyze participants’ memory performance: discriminability (A') and response bias (B''D). Discriminability reflects the ability to differentiate old and new items in the memory test, and individuals with fewer cognitive resources will show compromised discriminability [29]. Nevertheless, older adults [44] and Alzheimer’s disease patients [45] are more likely to exhibit a liberal response bias than the appropriate comparison group, which may serve as compensation for a decrease in discriminability. With SDT indices, we expected to differentiate the changes in discriminability and response bias under the different feedback conditions. In sum, the present study used a series of study-test trials to examine age-related differences in establishing and adjusting different memory strategies under various types of feedback (Study 1). Additionally, this study tested whether the memory strategy shaped by feedback would be retained after removing the feedback in subsequent tasks (Study 2). We also included a group of younger adults in a divided attention condition in Study 2 to investigate the role of attentional resources in age-related differences in memory strategy use.

## Study 1

In study 1, younger and older participants experienced a series of study-test trials under baseline, biased feedback and objective feedback conditions. Given that older adults have a natural tendency to rely more on gist-based processing [5], switching from a condition encouraging gist-based processing (i.e., BFB) to a condition encouraging item-specific processing (i.e., OFB) should be more difficult and effortful. Therefore, our interest was to examine whether OFB is sufficient to reshape the memory strategy when individuals previously relied on gist-based processing during BFB. In addition, by having participants experience baseline, BFB, and OFB in a fixed order, we observed how they adjusted their memory strategies relative to the previous condition. In the analysis, we compared only two successive conditions, i.e., baseline vs BFB and BFB vs OFB, to capture the dynamic adjustment of memory strategies as a function of feedback.

### Method

#### Participants

A total of 20 younger adults (nine women; age range = 19–25 years, *M* = 20.5 years, *SD* = 2.46) and 20 older adults (nine women; age range = 60–78 years, *M* = 67.2 years, *SD* = 5.12) participated in Study 1. Younger participants were university students, while older participants were recruited from the local community, and they all received RMB 60 (approximately USD 10) per hour for participation. All participants were screened for a history of alcoholism or substance abuse and medical conditions that might interfere with memory performance, including current or previous treatment for psychiatric illness, current treatment with psychoactive medication, drug toxicity, and primary degenerative brain disorders as well as brain damage sustained earlier from a known cause. The Mini-Mental State Examination (MMSE) was used to screen participants who might have dementia. All participants received a MMSE score above 28, and none were excluded for potential degenerative brain disease.

#### Ethics statement

The ethics review committee of the School of Psychological and Cognitive Sciences, Peking University approved the protocol details of our study, including the purpose, procedure, and materials. Participants provided written consent before taking part in this experiment, and they were fully debriefed at the end of the study.

#### Materials

A total of 120 word lists were drawn from a pool of Chinese associative word lists (developed for a study of younger people), and each list was composed of two critical lures and ten associates arranged in descending order of relatedness to the lure word. Besides the original critical lure, the most strongly associated item was selected as a second lure for each list in the test, and the two lures did not appear during the study. In a pilot study, ten older adults evaluated the word lists to ensure that there were no age-related differences in perceiving the relatedness between the associates and the critical lures. Ninety lists with the highest relatedness scores were selected from the original 120 word lists. Forty-five lists were randomly chosen to present for participants to study, while the other 45 were used as new items when testing participants’ memories. The lists used for the study and the test were counterbalanced among participants.

#### Design and procedure

The study used a 2 × 3 mixed design, that included a between-subjects factor “age group” [younger adults *vs* older adults] and a within-subjects factor “feedback” [baseline *vs* biased feedback (BFB) *vs* objective feedback (OFB)]. All participants followed the same experimental order, going through the baseline →BFB →OFB stages with a 24-hour interval after each feedback stage to minimize any fatigue effects.

There were five trials at each stage with a 30-second break between each trial. Thus, each participant completed a total of 15 study-test trials. Each trial consisted of two parts: the study and the test. In the study phase, participants were asked to view three word lists presented in a random order without a break in between. In each list, the associates were presented one at a time for 1,500 ms each in a descending order of relatedness. Then before taking the test, participants completed a simple addition filler task for 30 seconds (*e*.*g*., 12 + 65 = ?). In the test, 24 items appeared in a random order, including nine studied words (the 2nd, 5th, and 8th words of each studied list), six critical lures (two for each list), and nine new unrelated items (the 2nd, 5th, and 8th words of three non-studied lists). Participants were asked to judge whether an item was “*studied*” or “*new*” by pushing “F” or “J” on keyboard, and participants received feedback immediately after entering a response in the BFB and OFB stages.

During the baseline stage, participants received no feedback during the test. During the BFB stage, participants only received positive feedback (i.e., “Correct!”) when making a “studied” response to either a studied item or a critical lure, and no feedback was displayed after other responses to the test items in order to reinforce BFB’s enhancement for gist-based processing. In OFB, all of the responses received objective feedback in the test. If the response was accurate, “Correct!” in green would appear; otherwise, “Incorrect!” in red would appear.

### Result

In the formula proposed by Schacter et al. [41], “studied” responses to studied items (i.e., correct recognitions) reflect gist processing and/or item-specific processing or guessing; whereas “studied” responses to critical lures (i.e., false alarms to lures) were mostly based on gist processing or guessing, and “studied” responses to unrelated new items (i.e., false alarms to new items) were from guessing. Therefore, item-specific processing was assessed by calculating the discriminability (A') and response bias (B''D) in distinguishing between correct recognitions of the studied items and false alarms of critical lures [46]. Similarly, gist processing was assessed by calculating discriminability (A') and response bias (B''D) in making false alarms to critical lures from false alarms to new items (see Table 1 for the means of “studied” response percentage, A' and B''D of the three groups). Higher levels of A' represent greater discriminability or gist processing in the case of endorsing having remembered critical lures, while higher levels of B''D represent more liberal response bias.

**Table 1:** Mean Probability (standard error) of “studied” response to Studied Words, Lures, and Unrelated Words; Mean (standard error) of discriminability (A') and response bias (B''D).

		Old adults			Young adults		
		Baseline	BFB	OFB	Baseline	BFB	OFB
P(“studied”)	Studied	0.84	0.89	0.82	0.89	0.89	0.89
		(0.02)	(0.02)	(0.02)	(0.01)	(0.02)	(0.02)
	Lures	0.31	0.49	0.27	0.31	0.50	0.31
		(0.04)	(0.03)	(0.02)	(0.03)	(0.04)	(0.03)
	Unrelated	0.01	0.04	0.05	0.01	0.03	0.01
		(0.005)	(0.009)	(0.009)	(0.005)	(0.008)	(0.005)
A'	Gist	0.82	0.85	0.77	0.81	0.86	0.82
		(0.01)	(0.009)	(0.008)	(0.01)	(0.01)	(0.007)
	Item-specific	0.85	0.80	0.86	0.87	0.81	0.87
		(0.01)	(0.02)	(0.01)	(0.01)	(0.02)	(0.01)
B''D	Gist	0.90	0.78	0.67	0.88	0.82	0.92
		(0.04)	(0.05)	(0.05)	(0.04)	(0.05)	(0.03)
	Item-specific	-0.20	-0.46	-0.14	-0.38	-0.46	-0.43
		(0.07)	(0.05)	(0.05)	(0.06)	(0.07)	(0.07)

Note: A summary of younger and older adults’ mean percentages and standard error of “studied” response rate towards the studied items, critical lures, and unrelated new items, A' and B''D of gist and item-specific processing. The formula from Signal Detection Theory was modified and adopted to calculate discriminability (A') and response bias (B''D) of gist-based and item-specific processing. For discriminability (A'), H>FA, A' = 0.5+ [(H-FA)(1+FA -H)]/[4H(1-FA); H<FA, A' = 0.5-(FA-H)(1+FA-H)]/[4FA(1-H)]; H = FA, A' = 0.5. For response bias (B''D), H>FA, B''D = [H(1-H)-FA(1-FA)]/ [H(1-H)+FA(1-FA)]; H<FA, B''D = [FA(1-FA)-H(1-H)]/ [H(1-H)+FA(1-FA)]; H = FA, B''D = 0. In the calculation of indices on gist processing, “H” denoted “studied” response to critical lures, and “FA” denoted “studied” response to new unrelated items. In the calculation of indices on item-specific processing, “H” denoted “studied” response to studied items, and “FA” denoted “studied” response to critical lures.

Separate 2 × 3 mixed-model ANOVAs on discriminability (A') and response bias (B''D) were conducted. Separate repeated-measures ANOVAs assessed effects of feedback within age groups. All results were assessed by comparing the responses to the previous condition rather than to baseline.

#### Gist processing

**Fig 1:**
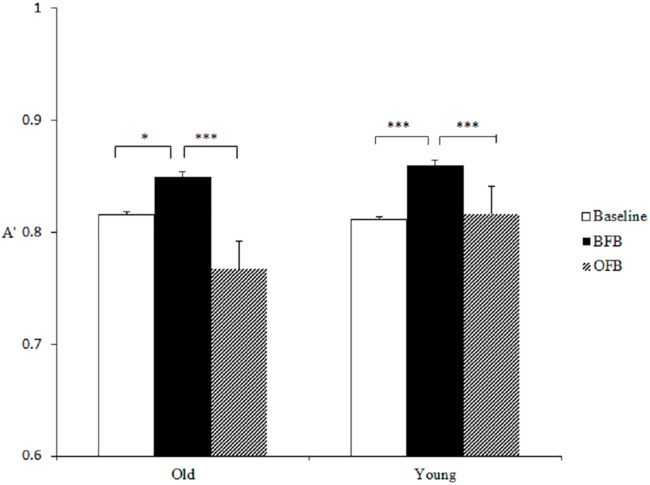
Two age groups’ discriminability (A') between critical lures and unrelated new items in Study 1. (For this and the following figures, the difference is marked with * if *p* < .05, ** if *p* < .01, and *** if *p* < .001. Errors bars indicate standard error.)

In the analysis of discriminability (A') of gist processing, the main effect of feedback was significant on A', *F* (2, 76) = 34.56, *p* < .001, *η²* = 0.48, The effect was qualified by a significant group × feedback interaction, *F* (2, 76) = 6.60, *p* = .002, *η²* = 0.15. By conducting separate repeated-measures ANOVAs for each age group, a significant effect of feedback was found among younger participants, *F* (2, 76) = 11.43, *p* < .001, *η²* = 0.39, with. *Post hoc* analysis using Bonferroni method (the same analysis was used in the following results) of means showed that compared with baseline (*M* = 0.81, *SE* = 0.01), A' increased under BFB (*M* = 0.86, *SE* = 0.01, *t* (19) = -.5.15, *p* < .001), and fell back under OFB (*M* = 0.82, *SE* = 0.007, compared with BFB, *t* (19) = 4.43, *p* = .001). A significant effect of feedback was also found among older adults, *F* (2, 76) = 28.89, *p* < .001, *η²* = 0.68. There was a trend that A' increased under BFB (*M* = 0.85, *SE* = 0.009, for baseline, *M* = 0.82, *SE* = 0.01, *t* (19) = -2.52, *p* = .10), and decreased under OFB (*M* = 0.77, *SE* = 0.008, *t* (19) = 8.80, *p* < .001), showing that the effects of BFB and OFB on gist-based processing were opposite of each other (see Fig 1). Comparing older adults and younger adults in each condition, older adults showed lower A' than did younger adults under OFB, *p* < .001. The results indicate that both younger and older adults benefitted from biased feedback to promote gist processing, and under objective feedback, older adults reduced the usage of gist to a greater level than did younger adults, which may suggest that they were more likely to rely on environmental supports to adjust their memory strategy.

**Fig 2:**
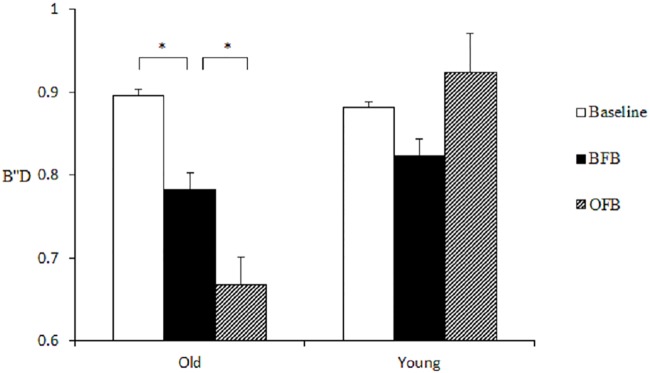
Two age groups’ response bias (B''D) between critical lures and unrelated new items in Study 1.

The analysis of response bias (B''D) revealed the significant main effects of feedback, *F* (2, 37) = 4.13, *p* = .024, *η²* = 0.18, and age, *F* (1, 38) = 5.539, *p* = .024, *η²* = 0.13, which were qualified by a significant interaction, *F* (2, 76) = 7.33, *p* = .001, *η²* = 0.16. By conducting separate repeated-measures ANOVAs for each age group, it was found that only among older adults was the effect of feedback significant, *F* (2, 76) = 9.30, *p* < .001, *η²* = 0.39; *post-hoc* analysis revealed that compared with baseline (*M* = 0.90, *SE* = 0.04), B''D showed a trend to decrease under BFB (*M* = 0.78, *SE* = 0.05, *t* (19) = 2.36, *p* = .087) and kept decreasing under OFB at a marginally significant level (*M* = 0.67, *SE* = 0.05, compared with BFB; *t* (19) = 2.36, *p* = .088). Among younger adults, no significant effect of feedback was found. This suggests that only older adults relaxed the response criterion under biased feedback but did not use OFB to change the use of the lax criterion (see Fig 2).

#### Item-specific processing

**Fig 3:**
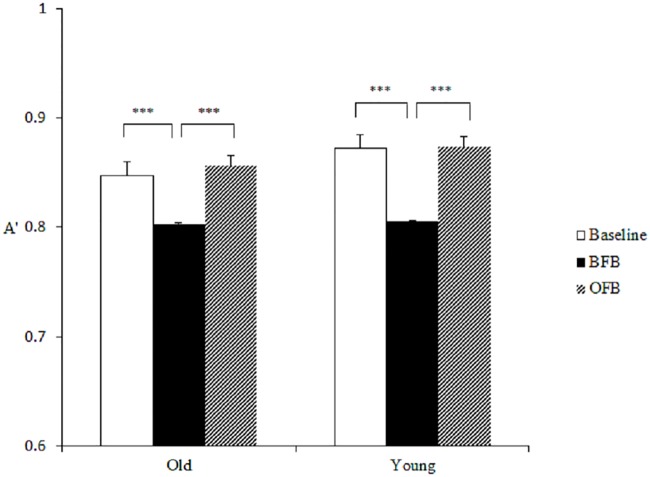
Two age groups’ discriminability (A') between studied items and critical lures in Study 1.

Item-specific processing showed a pattern opposite of gist processing across three feedback conditions. The only significant effect was the main effect of feedback on discriminability (A'), *F* (2, 76) = 24.34, *p* < .001, *η²* = 0.39. *Post hoc* analysis with Bonferroni procedure revealed that compared with the baseline (*M* = 0.86, *SE* = 0.01), A' decreased under BFB (*M* = 0.81, *SE* = 0.02, *t* (39) = 5.25, *p* < .001) and returned to the baseline level under OFB (*M* = 0.87, *SE* = 0.01, compared with BFB, *t* (39) = -6.57, *p* < .001). The findings indicated that biased feedback would compromise individuals’ item-specific processing, whereas objective feedback would enhance it (see Fig 3).

The analysis of B''D revealed a significant group × feedback interaction, *F* (2, 76) = 4.33, *p* = .028, *η²* = 0.102. Simple effects analysis found that the effect of feedback was significant only among older adults, *F* (2, 76) = 11.94, *p* < .001, *η²* = 0.58. Compared with baseline (*M* = -0.20, *SE* = 0.07), older participants’ response criterion became liberal under BFB (*M* = -0.46, *SE* = 0.05, *t* (19) = 3.12, *p* = .017) but was increased by OFB (*M* = -0.14, *SE* = 0.05, *t* (19) = -6.78, *p* < .001). No significant effect of feedback was found among younger adults.

Taken together, clear changes in memory-processing strategies following different types of feedback were found. Under biased feedback (i.e., encouraging “studied” responses based on gist information), both groups reduced item-specific processing and relied more heavily on gist-processing. However, when objective feedback was provided, both groups reduced gist processing and used more item-specific processing, especially older adults. Different from younger adults, older adults adjusted not only their discriminability but also their response criteria across three feedback stages, but once they adopted a liberal response bias for gist, they did not appear to adjust with objective feedback, as shown by the more liberal response bias under OFB. This may indicate a subjective compensation for age-related memory decline.

However, it was still unclear: (1) whether this feedback-shaped memory could be maintained in the subsequent tasks, and (2) what may cause the age-related difference in memory performance. To address these issues, Study 2 was conducted.

## Study 2

To address how older adults maintain memory strategies after feedback, in Study 2, we designed a condition with no feedback following the OFB stage to assess memory changes among younger and older adults. Moreover, a group of younger adults with divided attention was added to mimic the hypothesized attentional resources restrictions among older adults [47]. Although the aging process has various influences on cognition, the decline in attentional resources is hypothesized to lead to memory deficits [48]. As mentioned previously, due to limited attentional resources, older adults are less likely to use item-specific processing without environmental support. Previous studies found that distraction tasks weaken the inhibition effect of warning or repetitive learning on associative memory illusions and cause more memory errors among younger adults [7, 49, 50]. By testing the memory performance of older adults and a group of younger adults with restricted attentional resources, the underlying mechanism of aging memory might be better understood.

### Method

#### Participants

A total of 24 older adults (13 females; age range = 60–80 years, *M* = 70.3 years, *SD* = 3.12) and 44 undergraduate students (25 females; age range = 19–25 years, *M* = 21.3 years, *SD* = 2.89) took part in Study 2. All participants were recruited in the same way as those in Study 1, and MMSE was adopted as in Study 1 to screen people with early signs of dementia.

#### Ethics statement

As in Study 1, the ethics review committee of the School of Psychological and Cognitive Sciences, Peking University approved the protocol details of Study 2. Participants provided written consent before taking part in this experiment and were fully debriefed afterwards.

#### Design and procedure

We used the same word lists as in Study 1. The design was a 3 × 3 factorial design with feedback [baseline, objective feedback (OFB), post-feedback (PFB)] as the within-subjects factor and group [full-attention younger adults (FA young), *vs* divided-attention younger adults (DA young) *vs* older adults] as the between-subjects factor. All participants went through the experiment in the same order: baseline, OFB, and PFB. The baseline stage and PFB were identical to the baseline stage in Study 1, and the OFB was the same as the previous OFB. The interval between any two stages was 15 minutes, and participants were asked to play chess to relax during the breaks.

Forty-four younger participants were randomly assigned into the FA and DA groups (24 in FA). For the DA young group, an oddball paradigm was adopted to generate distraction throughout the study phases, in which a total of 20 sound signals were presented at 500 ms before and after each study item, 10–20% of which had a high-pitched tone. Participants were asked to press the “SPACE” key whenever they heard a high-pitched signal. When participants missed responding to more than three high-pitched signals, they would be required to redo the trial (no participant reached this criterion).

### Results

We used the same indices to measure gist and item-specific processing as in Study 1. Separate 3 × 3 mixed model ANOVAs were conducted on A' and B''D (see Table 2).

**Table 2:** Mean Probability (standard error) of “studied” response to Studied Words, Lures, and Unrelated Words; Mean (standard error) of discriminability (A') and response bias (B''D).

		Old adults			FA young			DA young		
		Baseline	OFB	PFB	Baseline	OFB	PFB	Baseline	OFB	PFB
P(“studied”)	Studied	0.91	0.87	0.90	0.95	0.91	0.94	0.93	0.89	0.93
		(0.02)	(0.02)	(0.01)	(0.01)	(0.01)	(0.01)	(0.02)	(0.02)	(0.01)
	Lures	0.39	0.21	0.42	0.42	0.17	0.14	0.42	0.20	0.38
		(0.03)	(0.02)	(0.03)	(0.03)	(0.02)	(0.02)	(0.05)	(0.03)	(0.04)
	Unrelated	0.03	0.02	0.03	0.01	0.002	0.01	0.02	0.01	0.02
		(0.01)	(0.01)	(0.01)	(0.004)	(0.002)	(0.005)	(0.01)	(0.01)	(0.01)
A'	Gist	0.82	0.80	0.84	0.85	0.77	0.72	0.83	0.78	0.83
		(0.01)	(0.03)	(0.01)	(0.007)	(0.02)	(0.03)	(0.02)	(0.02)	(0.01)
	Item-specific	0.86	0.90	0.83	0.86	0.93	0.94	0.85	0.91	0.86
		(0.01)	(0.01)	(0.01)	(0.01)	(0.009)	(0.009)	(0.02)	(0.01)	(0.02)
B''D	Gist	0.81	0.79	0.83	0.91	0.98	0.88	0.85	0.81	0.85
		(0.05)	(0.05)	(0.04)	(0.03)	(0.02)	(0.05)	(0.05)	(0.10)	(0.05)
	Item-specific	-0.53	-0.20	-0.44	-0.71	-0.25	-0.17	-0.59	-0.10	-0.53
		(0.08)	(0.11)	(0.05)	(0.05)	(0.11)	(0.13)	(0.08)	(0.09)	- (0.06)

Note: A summary of the three groups’ mean percentages and standard error of “studied” response rate towards studied items, critical lures, and unrelated new items. The means and standard error of discriminability (A') and response bias (B''D) of gist-based processing (by distinguishing critical lures and unrelated new items) and item-specific processing (by distinguishing critical lures and studied items) were also included.

#### Gist processing

**Fig 4:**
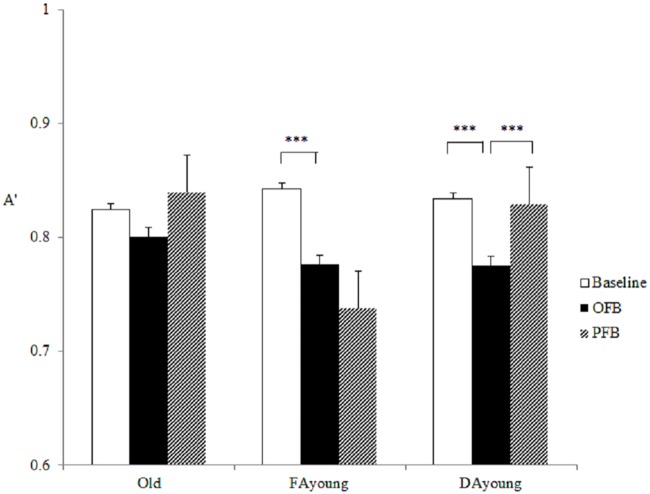
Three groups’ discriminability (A') between critical lures and unrelated new items in Study 2.

The main effect of feedback was significant on A' of gist processing, *F* (2, 130) = 8.042, *p* = .001, *η*² = 0.110, and the main effect of group was also significant, *F* (2, 65) = 3.715, *p* = .03, *η*² = 0.103. The results were qualified by a significant Group × Feedback interaction, *F* (4, 130) = 6.337, *p* < .001, *η²* = 0.163. We probed this interaction with separate repeated measures ANOVAs in each group. For FA young, the effect of feedback was significant, *F* (2, 92) = 13.76, *p* < .001, *η²* = 0.43. Compared with baseline (*M* = 0.85, *SE* = 0.007), gist processing was reduced under OFB (*M* = 0.77, *SE* = 0.02, *t* (23) = 4.41, *p* = .001) and maintained at that level under PFB (*M* = 0.72, *SE* = 0.03, *t* (23) = 2.31, *p* = .09), thereby indicating that FA young had the ability to adjust their memory strategy under feedback and to retain it afterwards. For DA young, the main effect of feedback was also significant, *F* (2, 92) = 4.26, *p* = .017, *η²* = 0.22. Compared to baseline (*M* = 0.83, *SE* = 0.02), A' was significantly reduced under OFB (*M* = 0.78, *SE* = 0.02, *t* (19) = 2.70, *p* = .04), but it increased under PFB (*M* = 0.83, *SE* = 0.01, compared with OFB, *t* (19) = -3.32, *p* = .01), showing that DA young were not able to maintain the newly established memory strategy when the feedback was gone. For older adults, the effect of feedback on A’ was not significant. The findings suggest younger participants reduced gist processing with immediate objective feedback, but that only FA young group preserved using the strategy (see Fig 4).

B''D of gist processing only showed a significant group effect, *F* (2, 65) = 3.52, *p* = .035, *η²* = 0.098. *Post hoc* analysis with Bonferroni procedure showed that FA young adopted a stricter response criterion *(M* = 0.92, *SE* = 0.03) than older adults (*M* = 0.81, *SE* = 0.05, *t* (46) = -2.84, *p* = .043), but the difference from DA young (*M* = 0.84, *SE* = 0.07) was not significant, *p* = .16, and there was no significant difference between older adults and DA young.

#### Item-specific processing

**Fig 5:**
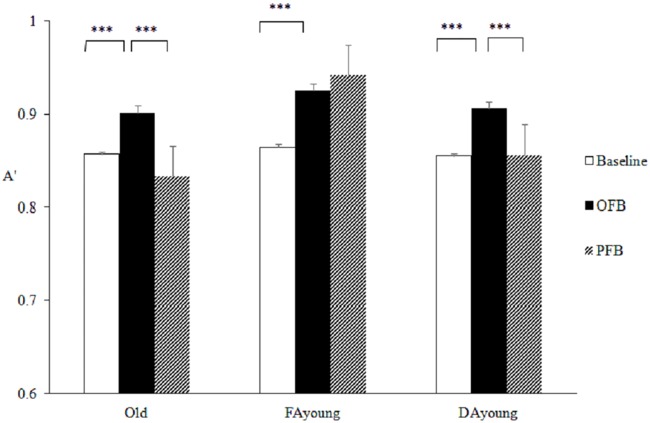
Three groups’ discriminability (A') between studied items and critical lures in Study 2.

There was a significant main effect of feedback on the discriminability (A') of item-specific processing, *F* (2, 130) = 19.002, *p* < .001, *η²* = 0.226. For OFB (*M* = 0.91, *SE* = 0.01), and the group effect was also significant, *F* (2, 65) = 6.83, *p* = .002, *η²* = 0.174. Meanwhile, the effects were qualified by a significant interaction, *F* (4, 130) = 7.626, *p* < .001, *η²* = 0.19. By conducting separate repeated measures ANOVAs in each group, we found that FA young showed a significant effect of feedback, *F* (2, 92) = 17.71, *p* < .001, *η²* = 0.49. Compared with the baseline (*M* = 0.86, *SE* = 0.01), A' significantly increased under OFB (*M* = 0.93, *SE* = 0.009, *t* (23) = -5.50, *p* < .001) and remained stable under PFB (*M* = 0.94, *SE* = 0.009, *t* (23) = -1.71, *p* = .3), thereby suggesting that the feedback-enhanced item-specific processing could be maintained among FA young. For older adults, the main effect of feedback was also significant, *F* (2, 92) = 12.73, *p* < .001, *η²* = 0.40. Compared with the baseline (*M* = 0.86, *SE* = 0.01), A' increased under OFB (*M* = 0.90, *SE* = 0.01, *t* (23) = -3.87, *p* = .002) but fell back on PFB (*M* = 0.83, *SE* = 0.01, *t* (23) = 4.55, *p* < .001), indicating a temporary effect of feedback. The DA young showed a similar pattern with older adults, as the main effect of feedback was significant, *F* (2, 84) = 7.64, *p* = .001, *η²* = 0.44. A' increased under OFB (*M* = .91, *SE* = .06, *t* (19) = -3.32, *p* = .01) compared to baseline (*M* = 0.86, *SE* = .08) and dropped under PFB compared with OFB, *M* = .86, *SE* = .10, *t* (19) = 3.65, *p* = .006; this finding suggests that the enhancement of item-specific processing driven by feedback actually relies on environmental support (see Fig 5).

**Fig 6:**
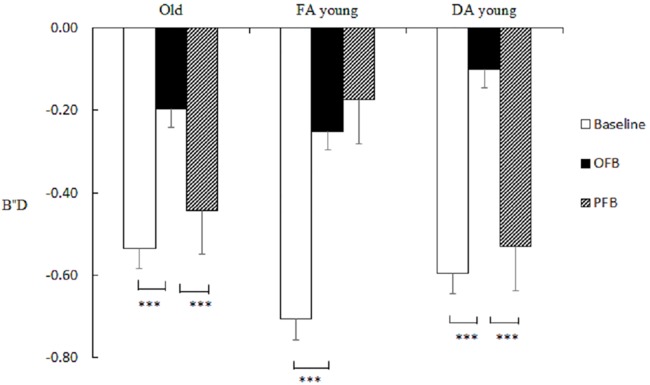
Three groups’ response bias (B''D) between studied items and critical lures in Study 2.

On B''D, the feedback effect was significant, *F* (2, 130) = 22.95, *p* < .001, *η²* = 0.26, and was qualified by a significant interaction effect, *F* (4, 130) = 3.98, *p* = .004, *η²* = 0.11. Simple effect analysis revealed that the feedback effect was significant among FA young, *F* (2, 88) = 11.72, *p* < .001, *η²* = 0.35, with higher B''D under OFB (*M* = -0.25, *SE* = 0.11) than the baseline (*M* = -0.71, *SE* = 0.05), *t* (23) = -4.03, *p* = .002, that was maintained at that level under PFB (*M* = -0.17, *SE* = 0.13, *p* = .55). The feedback effect was also significant for older adults, *F* (2, 88) = 3.73, *p* = .028, *η²* = 0.16, but compared with the baseline (*M* = -0.53, *SE* = 0.08), B''D only increased under OFB (*M* = -0.20, *SE* = 0.11, *t* (23) = -3.29, *p* = .009), whereas it decreased under PFB (*M* = -0.44, *SE* = 0.05, compared with OFB, *t* (23) = 2.43, *p* = .05). The main effect of feedback was also significant among DA young, *F* (2, 88) = 9.53, *p <* .001, *η²* = 0.34, with a pattern that replicated that of older adults across three stages. B''D significantly increased under OFB (*M* = -0.10, *SE* = 0.09) compared with the baseline (*M* = -0.59, *SE* = 0.08, *t* (19) = -4.63, *p* < .001), and decreased under PFB (*M* = -0.53, *SE* = 0.06) compared with OFB, *t* (19) = 4.78, *p* < .001. The results showed unlike FA young, older adults and DA young were capable of only applying a stricter response criterion with immediate feedback (see Fig 6.).

Taken together, the immediate objective feedback helped all three groups to successfully inhibit false memories by reducing gist-based processing and facilitating item-specific processing. However, except for FA young, the OFB-increased discriminability and response criteria were not retained when the feedback was removed.

## General Discussion

By investigating dynamic changes in memory processing with STD indices, our findings clarified how older and younger adults adjusted and maintained memory strategy after receiving different types of feedback. In Study 1, we found both older adults and younger adults were able to enhance gist processing with biased feedback and switch to item-specific processing when objective feedback was provided. In Study 2, we examined the ability to maintain the memory strategy shaped by feedback among younger and older adults. We found that reduction in attentional resources played a critical role in preventing individuals from retaining the feedback-shaped memory strategy after the objective feedback was removed. The findings suggest that age-related cognitive resource decline is a key reason why older adults spontaneously rely more on gist-based processing, but that sufficient environmental support (e.g., immediate objective feedback) can ameliorate this decline and enhance item-specific processing.

### Adjusting Encoding and Monitoring with Feedback

As mentioned above, few studies have tested how feedback influences gist and verbatim encoding. Most previous studies on feedback provided only quantitative evaluation of memory performance (e.g., “80% of the responses were ‘correct’”). In contrast, the present study focused on the cumulative effect of immediate feedback in the subsequent encoding and monitoring of memories. Receiving “Correct!” feedback may increase response tendencies and confidence, thus encouraging participants to make similar responses, while getting “Incorrect!” feedback would prompt them to avoid making the same mistake again. The two sets of indices to capture gist processing and item-specific processing clearly showed the relation between feedback, processing type, and age. When receiving positive feedback for recognizing associative items (i.e., false recognition of critical lures), gist processing was reinforced, which made younger participants respond in the same way as older adults. On the contrary, when receiving objective feedback emphasizing the subtle perceptual and sensory distinction between the studied items and critical lures, even the older adults adjusted their memory strategy and reduced false memories.

In addition, the feedback-shaped focus of encoding may further affect monitoring during retrieval. The encoding strategy under biased feedback left less item-specific information to be utilized for monitoring, while objective feedback left more. Therefore, according to the activation/monitoring framework, with more item-specific processing, individuals can better monitor their memory judgments, thus increasing accuracy. Specifically, objective feedback could improve disqualifying monitoring, which refers to rejection based on the recall of inconsistent information [11]. Therefore, with “Correct/Incorrect” feedback in the prior test, individuals would be more cautious when making their responses. For instance, they may recall that “last time when I made an ***studied*** response with hesitation, I was told ‘incorrect!’,” thus, suppressing “studied” responses towards critical lures and adjusting their memory strategy. These findings are consistent with previous work that found, with feedback in an original test, participants corrected the originally incorrect “studied/not studied” response in a retest. Their EEG recordings also indicated that monitoring was involved in this process. Specifically, the brain activity in making a correction were similar to that of monitoring in the lateral prefrontal cortex [1].

Taken together, our findings build upon and extend previous work by showing that: (1) the retrieval environment impacted participants’ subsequent memory processing; (2) feedback affected both discriminability and response criteria; and (3), most importantly, feedback affected not only younger adults but also older adults, which indicates a feasible and effective memory training for aged groups.

### Adaptive Nature of Age-related Memory Strategy

The findings also suggest that older adults’ item-specific processing relies heavily on the accessibility of environmental supports. As mentioned in the introduction, with repeated study, older adults showed an increase in associative memory illusion in the first few trials and a decrease later; whereas, younger adults showed decreased false alarms throughout [28]. This may indicate that older adults automatically prioritize gist-based processing, which would lead to more associative memory illusions, and only with extra environmental support would they process item-specific information and reduce their false memories. Our results were consistent with this. We found that when the support (e.g., feedback) was removed, older adults as well as younger adults with divided attention switched to gist processing from distinctive processing. Our results supported that a reduction in attentional resources could cause underlying age-related differences in false memory. The increase in gist processing after feedback was removed among divided-attention younger adults and older adults is consistent with gist processing being a default option of a compromised aging memory system, while processing item-specific information requires environmental supports (e.g., feedback) for these individuals. Without support, they automatically choose gist processing. As mentioned earlier, gist processing is more critical for retaining essential information, which may underscore that memory illusions are a by-product of the adaptive feature of memory.

The SOC theory [18] predicts that when cognitive functions are limited, older individuals can adjust their strategies to achieve goals and, if necessary, even adjust cognitive goals [19]. In line with this notion, our findings demonstrate that older adults adjust their memory strategy to better store essential information, such that they use gist processing spontaneously as a dominant strategy, while the initiation and maintenance of item-specific processing requires environmental help. In other words, the trade-off between memory accuracy and essential information may reflect the adaptive nature of aging memory strategies. By prioritizing the goal of preserving critical information, older adults perform better to maintain cognitive resilience when facing declines caused by the aging brain.

### Limitations and Future Direction

The present study showed that older adults as well as younger adults can use feedback to adjust their memory strategies. Older adults, who have limited attentional resources, failed to maintain the formed memory strategy when feedback was removed. However, there are a few limitations that should be noted when conducting future studies. First, the ratio difference in positive feedback between BFB and OFB could potentially influence participants’ response biases. Thus, in future studies, similar ratios of positive feedback should be provided across feedback conditions (e.g., in BFB, display both “correct” feedback to the false alarms of critical lures and “incorrect” feedback to the rejections of them) in order to match the ratio of positive feedback under OFB. Second, although the results showed that older adults and younger adults with divided attention were unable to retain the feedback-shaped memory strategy when the feedback was removed, this is not sufficient evidence to claim that they could not maintain the memory strategy with stronger environmental support (e.g., longer-term feedback, adding rewards or meanings). The answers to this issue will provide insights for caregiving and memory training for older adults and populations with memory impairment. In addition, in our study it is not clear whether the effects of feedback mainly occur during encoding, retrieval, or post-retrieval monitoring, or whether multiple processes are involved. Meanwhile, distraction in a different stage (i.e., encoding or retrieval) may also lead to different effects regarding how feedback influences memory strategies. Furthermore, future studies may adopt neuroimaging techniques to better understand the neural mechanisms underlying the effects of feedback.

In summary, the current study extends previous literature by showing the adaptive nature underlying the dynamic changes of memory strategies with feedback. It also supports that age-related differences in establishing and maintaining memory strategies are due to cognitive declines. Specifically, both younger and older adults have the ability to regulate memory performance under feedback, but older adults’ reduction in attentional resources hinders maintenance of their feedback-shaped strategy. In other words, in accordance with the accessibility of environmental support, older adults can flexibly adjust their memory strategy, but without external assistance they will spontaneously employ a more economical memory strategy, i.e., gist-based processing.

## Supporting Information

S1 file Experiment Data of memory recognition. (XLSX)
